# Surveillance for West Nile Virus in Dead Wild Birds, South Korea, 2005–2008

**DOI:** 10.3201/eid1702.100551

**Published:** 2011-02

**Authors:** Jung-Yong Yeh, Hyun-Ju Kim, Jin-Ju Nah, Hang Lee, Young-Jun Kim, Jin-San Moon, In-Soo Cho, In-Soo Choi, Chang-Seon Song, Joong-Bok Lee

**Affiliations:** Author affiliations: National Veterinary Research and Quarantine Service, Gyeonggi-do, South Korea (J.-Y. Yeh, H-J. Kim, J.-J. Nah, J.-S. Moon, I.-S. Cho);; Seoul National University, Seoul, South Korea (H. Lee, Y.-J. Kim);; Konkuk University, Seoul (I.-S. Choi, C.-S.Song, J.-B. Lee)

**Keywords:** West Nile Virus, viruses, wild birds, zoonoses, surveillance, South Korea, dispatch

## Abstract

To investigate the possibility of West Nile virus (WNV) introduction into South Korea, the National Veterinary Research and Quarantine Service has conducted nationwide surveillance of WNV activity in dead wild birds since 2005. Surveillance conducted during 2005–2008 found no evidence of WNV activity.

Wild birds are considered the principal hosts of West Nile virus (WNV). In the United States, surveillance of birds for WNV is used to quickly detect outbreaks and take action against its spread. The sampling of sick or dead birds can indicate WNV in a region before human and equine cases occur (*1*). This approach is considered the most effective method for detecting WNV in a specific region. During 1999, mass deaths among wild birds indicated the emergence and rapid spread of WNV in North America.

Although WNV has not yet been detected in South Korea, the perceived threat of its arrival has been highlighted by reports of WNV infection in a dead cinereous vulture (*Aegypius monachus*) in the Vladivostok region of Russia, which is adjacent to the Korean peninsula (*2*), and in several samples from cinereous vultures and cattle egrets (*Bubulcus ibis*) in the Russian Far Eastern Region during 2002–2004 (*3*). A variety of migratory birds, such as Mandarin ducks (*Aix galericulata*), cinereous vultures, bean geese (*Anser fabalis*), and white-fronted geese (*Anser albifrons*), fly from Russia to South Korea during the winter for the breeding season (*4**–**6*). Furthermore, Saito et al. recently reported that test results on several migrating birds captured in Japan were positive for flavivirus antibodies (*7*). This finding suggests that the threat of WNV in South Korea is increasing because many migratory birds share flyways over South Korea and Japan (*8*). Therefore, spread of the virus by migratory birds from WNV-infected areas, such as Russia, into uninfected hosts throughout the Korean peninsula is likely.

## The Study

A wide variety of bird species from all regions of South Korea were tested, and particular attention was paid to susceptible species and birds with neurologic signs. Carcasses of wild birds submitted to the Conservation Genome Resource Bank for Korean Wildlife, Seoul National University, Seoul, South Korea, were used for this study. The study also included samples from dead wild birds submitted to the Animal Disease Diagnostic Center of the National Veterinary Research and Quarantine Service of the Ministry of Food, Agriculture, Forestry and Fisheries of South Korea.

Investigation focused on the presumed peak period of mosquito vector activity (April–October) and included samples from dead wild birds. A total of 715 wild birds (belonging to 72 species) from all regions of South Korea were found dead and were examined during 2005–2008. All carcasses underwent postmortem examination, during which samples were obtained for diagnosis. In 2005, a total of 51 samples were tested; 167 samples were tested in 2006, 239 in 2007, and 258 in 2008. Taxonomic families of the collected birds and their migratory status are shown in Table A1. Samples from *Ae. monachus*, *A. fabalis*, and *A. albifrons* birds, which are known to migrate from the Russian Eastern Region to South Korea (*4**,**5*), were included. Samples of dead wild birds such as *Corvidae* spp. and raptors (*Accipitridae* and *Strigidae* spp.), which have been identified as potential sources of WNV for resident birds (*9**,**10*), were also included.

Carcasses were subjected to necropsy, and brains and kidneys were obtained. Organs were homogenized in phosphate-buffered saline (10% suspension) and centrifuged. Ten 50% tissue culture infectious doses of a stock WNV were used as a control for antigen detection. WNV RNA in samples was investigated by reverse transcription–PCR with primers (Table). Information on the RNA extraction and the reverse transcription–PCR used is available in the Technical Appendix.

During 2005–2008, we analyzed 1,309 organ samples (639 brain and 670 kidney) from dead birds for WNV RNA. WNV was not detected in these samples. Diagnostic examination of wild birds as a part of the nationwide surveillance has not detected patterns or clusters of birds with evidence of neurologic disease or viral encephalitides suggestive of WNV infection. Several cases of mass die-offs among wild birds were the result of chemical poisoning (*11*).

## Conclusions

Our surveillance of wild birds conducted during 2005–2008 supports the hypothesis that WNV has not reached South Korea and corroborates findings of previous reports. In a study conducted at the National Institute of Health, Korea Centers for Disease Control and Prevention, 2,275 pools of mosquitoes were tested for WNV RNA; results for all samples obtained during 2006–2008 were negative (*12*). The study reported that 27 cerebrospinal fluid samples and 57 serum specimens obtained from patients who were suspected of having Japanese encephalitis and dengue fever were also negative for WNV. In another surveillance study of mosquitos and crows in Japan, a country near South Korea, no WNV RNA was detected. This study included mosquitoes obtained in a park in Tokyo during 2002–2006 and 329 captured or dead crows obtained during1994–2006 (*13*). In addition, antibodies against WNV antibodies were not detected in 18 crows sampled during 1995–2003. The first human WNV infection in Japan was confirmed in a person who returned from the United States in 2005 (*14*). However, no indigenous human or equine cases have been reported.

Although our surveillance found no evidence of WNV in South Korea, WNV could be introduced into this country in the near future. Moreover, several species of mosquitoes with the ability to transmit WNV have been identified in South Korea. Turell et al. reported that mosquitoes captured in Paju County, Gyeonggi Province, South Korea, were highly susceptible to WNV infection when they fed on viremic chickens (*15*).

Introduction of WNV into South Korea would undoubtedly become a major public health problem. An outbreak similar to the one that occurred in New York during 1999 could result in the disease becoming endemic to the country. Continued surveillance of dead wild birds is essential to enable prompt detection of WNV. Additionally, WNV surveillance programs in South Korea should continue to examine cases of viral encephalitis in horses and mass deaths among birds. Temperature increases caused by climate change should also be taken into account, and vigilant monitoring of emerging arboviruses, in addition to WNV, will be required. Finally, increased cooperation between the government and other agencies, such as wildlife conservation organizations and horse-racing authorities, is needed for early detection of WNV disease and development of effective veterinary and public health strategies.

## Supplementary Material

Technical Appendix Reverse Transcription PCR Methods.

## Appendix

**Table A1:** Migration status (seasonality) and abundance of 715 dead wild birds (72 species) with West Nile virus infection, South Korea, 2005–2008

Family, common name	Species	No. samples*	Migration status†
Accipitridae			
Cinereous vulture	*Aegypius monachus*	1	W3, RV3
Common buzzard	*Buteo buteo*	8	P3, W3, SV3
Eurasian sparrowhawk	*Accipiter nisus*	1	P3, RV2
Common kingfisher	*Alcedo atthis*	3	S2, R(m)5
Black-capped kingfisher	*Halcyon pileata*	2	S3
Anatidae			
Baikal teal	*Anas formosa*	6	W1, SV3
Pintail	*Anas acuta*	2	P2, W2
White-fronted goose	*Anser albifrons*	6	P1, W2, SV2
Common teal	*Anas crecca*	7	W2, RV1
Parrot	*Lorius domicella*	1	Exotic
Mandarin duck	*Aix galericulata*	2	R(m)3, W3
Mallard	*Anas platyrhynchos*	60	P1, W1, R4
Bean goose	*Anser fabalis*	2	P1, W2, SV2
Spot-billed duck	*Anas poecilorhyncha*	16	P1, W1, R2
Ardeidae			
Striated heron	*Butorides striatus*	3	S3
Great egret	*Casmerodius albus*	2	W3, SV1
Little egret	*Egretta garzetta*	8	S2, W4
Gray heron	*Ardea cinerea*	9	S3, W3
Great egret	*Egretta alba*	4	S2, WV1
Intermediate egret	*Egretta intermedia*	1	S3
Black-crowned night heron	*Nycticorax nycticorax*	5	S3, R4
Buff-backed heron, cattle egret	*Bubulcus ibis*	10	S2
Caprimulgidae			
Gray nightjar	*Caprimulgus indicus*	5	P3, S3
Ciconiidae			
Oriental white stork	*Ciconia boyciana*	1	W5, SV3
Columbidae			
Rufous turtle dove	*Streptopelia orientalis*	19	R1, P3
Hill pigeon	*Columba rupestris rupestris*	39	R5
Feral rock pigeon	*Columba livia*	3	R(m)2
Coraciidae			
Broad-billed roller	*Eurystomus orientalis*	3	P3, S3
Corvidae			
Black-billed magpie	*Pica pica*	96	R(m)1
Azure-winged magpie	*Cyanopica cyana*	1	R(m)2
Jay	*Garrulus glandarius*	6	R(m)1
Jungle crow	*Corvus macrorhynchos*	3	R(m)2
Cuculidae			
Oriental cuckoo	*Cuculus saturates*	1	S2
Common cuckoo	*Cuculus canorus*	1	S1
Emberizidae			
Rustic bunting	*Emberiza rustica*	2	P1, W1
Falconidae			
Eurasian hobby	*Falco subbuteo*	10	S3
Common kestrel	*Falco tinnunculus*	11	R(m)3, S2
Fringillidae			
Eurasian siskin	*Carduelis spinus*	1	P1, W1, SV3
Gray-capped greenfinch	*Carduelis sinica ussuriensis*	1	R(m)1, W2
Gaviidae			
Red-throated diver	*Gavia stellata*	1	P3, W3
Hirundinidae			
House swallow	*Hirundo rustica*	1	P1, S1, WV3
Laridae			
Black-tailed gull	*Larus crassirostris*	2	S2, W2
Herring gull	*Larus argentatus*	1	W2, RV1
Muscicapidae			
Blue-and-white flycatcher	*Cyanoptila cyanomela*	1	P2, S2
Oriolidae			
Black-naped oriole	*Oriolus chinensis*	2	P2, S2
Paradoxornithidae			
Vinous-throated parrotbill	*Paradoxornis webbiana*	1	R(m)1
Paridae			
Great tit	*Parus major*	2	R(m)1
Passeridae			
Tree sparrow	*Passer montanus*	18	P3, W3, R5
Phasianidae			
Golden pheasant	*Chrysolophus pictus*	1	Exotic
Ring-necked pheasant	*Phasianus colchicus*	39	R1
Chicken	*Gallus gallus domesticus*	1	R1
Korean black chicken	*Gallus gallus var. domesticus*	1	R1
Picidae			
Great spotted woodpecker	*Dendrocopos major*	1	R(m)2
Japanese pigmy woodpecker	*Dendrocopos kizuki*	1	R1
Green woodpecker	*Picus viridus*	1	R(m)3
Procellariidae			
Streaked shearwater	*Calonectris leucomelas*	1	S2
Pycnonotidae			
Brown-eared bulbul	*Hypsipetes amaurotis*	11	R(m)1, S3, W3
Rallidae			
Coot	*Fulica atra*	1	W3, R4
Moorhen	*Gallinula chlororpus*	1	S4, R5
Scolopacidae			
Woodcock	*Scolopax rusticola*	6	P4, W5
Whimbrel	*Numenius phaeopus variegatus*	1	P3, WV3
Strigidae			
Eurasian scops owl	*Otus scops stictonotus*	33	P3, S3
Brown hawk owl	*Ninox scutulat*	37	P4, S4
Eurasian eagle owl	*Bubo bubo*	20	R(m)4
Tawny owl	*Strix aluco*	1	R4
Long-eared owl	*Asio otus*	2	P5, W5
Collared scops owl	*Otus lempiji*	7	R(m)3, W4
Sturnidae			
Gray starling	*Sturnus cineraceus*	1	W2, R(m)2
Turdidae			
Gray-backed thrush	*Turdus hortulorum*	1	P3, S3
White`s thrush	*Zoothera dauma*	13	S2, W4
Zosteropidae			
Japanese white-eye	*Zosterops japonica japonica*	1	R(m)3, P4
Unidentified		142	
Total		715	

*Samples were received from natural heritage centers, wildlife rescue organizations, and private veterinary practices. †Letters are used in a wide range of combinations to suggest a species’ seasonality. R, resident; R(m), resident and partial migrant; P, passage migrant (i.e., spring, autumn, or both); W, winter visitor; S, summer visitor or summer resident. Numbers (1–5) are used to express estimated abundance since 1980: 1, numerous (>100,000 records or individuals); 2, rather common/locally common (10,000–100,000 records or individuals); 3, fairly common (1,000–<10,000 records or individuals); 4, uncommon or rather local (100–<1,000 records or individuals); 5, scarce or very local (recorded annually, with ≥100 records from 1980 to the present time and <100 records estimated to occur annually). For less regularly recorded species, V followed by a number (1–5) indicates all known records (from 1980 to the present time): V1, probable annual (25–99 records or individuals); V2, recorded scarcely annually, or less than annually (10–<25 records or individuals); V3, ≥10 records, n); V4, species last recorded >10 years ago; V5, species added to the Birds Korea Checklist since the past update (starting in October 2007). On occasion, these codes are also used with a prefix (e.g., W, S) to indicate that more abundant species also occur more rarely in a given season (between 1980 and the present time). For example, S3, WV3 indicates that a species that is fairly common in summer has also been recorded <10 times in mid-winter between 1980 and the present time. This manner of measuring migratory status (seasonality) and abundance is followed by The Birds Korea Checklist: 2009 (*6*).

**Table:** Oligonucleotide primers used for reverse transcription–PCR of West Nile virus in dead wild birds, South Korea, 2005–2008

Primer	Sequence, 5’ →3’	Orientation*	Genome position†	Product size, bp
WN233	TTGTGTTGGCTCTCTTGGCGTTCTT	S	233	408
WN640	CAGCCGACAGCACTGGACATTCATA	AS	640	408
AmWN1401	ACCAACTACTGTGGAGTC	S	1401	445
AmWN1845	TTCCATCTTCACTCTACACT	AS	1845	445
AmWN1485	GCCTTCATACACACTAAAG	S (nested PCR)	1485	248
AmWN1732	CCAATGCTATCACAGACT	AS (nested PCR)	1732	248

*S, sense; AS, antisense. †Genbank accession no. NC_009942.

## References

[1] Eidson M, Kramer L, Stone W, Hagiwara Y, Schmit K. Dead bird surveillance as an early warning system for West Nile virus. Emerg Infect Dis. 2001;7:631–5. 10.3201/eid0704.010405 11585524 PMC2631773

[2] Loktev VB. West Nile virus, vulture—Russia (VLADIVOSTOK): ProMED-MAIL; 2004 [cited 2010 Dec 21]. http://www.promedmail.org

[3] Ternovoĭ VA, Protopopova EV, Surmach SG, Gazetdinov MV, Zolotykh SI, Shestopalov AM, The genotyping of the West Nile virus in birds in the Far Eastern Region of Russia in 2002–2004 [in Russian]. Mol Gen Mikrobiol Virusol. 2006;4:30–5. 17094656

[4] Jin S-D, Baek U-K. Research on wintering of *Aegypius monachus* in Korea. Mun Hwa Jae. 2009;42:62–71.

[5] Kim J, Park J, Yoo B, Rhee D. The migration route and monitoring of the migratory birds in Korea. In: Wildlife Biology. Inchon (South Korea): National Institute of Environmental Research; 2002. Report 24:153–64.

[6] Moores N, Park J-G, Kim A. The birds Korea checklist: 2009 [cited 2010 Dec 21]. http://www.birdskorea.org

[7] Saito M, Osa Y, Asakawa M. Antibodies to flaviviruses in wild ducks captured in Hokkaido, Japan: risk assessment of invasive flaviviruses. Vector Borne Zoonotic Dis. 2009;9:253–8. 10.1089/vbz.2008.0111 19514809

[8] Lee W-S, Gu T-H, Park J-Y. A field guide to the birds of Korea. Seoul (South Korea): LG Foundation; 2005.

[9] Komar N, Langevin S, Hinten S, Nemeth N, Edwards E, Hettler D, Experimental infection of North American birds with the New York 1999 strain of West Nile virus. Emerg Infect Dis. 2003;9:311–22. 12643825 10.3201/eid0903.020628 PMC2958552

[10] Nemeth N, Gould D, Bowen R, Komar N. Natural and experimental West Nile virus infection in five raptor species. J Wildl Dis. 2006;42:1–13. 16699143 10.7589/0090-3558-42.1.1

[11] Genome Resource Bank for Korean Wildlife. 2009 [cited 2010 Dec 22]. http://www.cgrb.org/index_e.htm

[12] Han M-G, Lee H-I, Lee C-S, Lee W-G, Jeong Y-E, Cho J-E, Surveillance of West Nile viruses from 2006 to 2008, Korea: no evidence of infection. The Microbiological Society of Korea’s International Symposium. May 28–30, 2009. Jeju Island, Seoul (South Korea): The Microbiological Society of Korea; 2009. p. 237.

[13] Tabei Y, Hasegawa M, Iwasaki N, Okazaki T, Yoshida Y, Yano K. Surveillance of mosquitoes and crows for West Nile virus in the Tokyo metropolitan area. Jpn J Infect Dis. 2007;60:413–6. 18032852

[14] Koizumi K, Nakajima Y, Matsuzaki M, Koido N, Ohsone Y, Lim CK, First report of West Nile fever in Japan [in Japanese]. Kansenshogaku Zasshi. 2006;80:56–7. 16519126 10.11150/kansenshogakuzasshi1970.80.56

[15] Turell MJ, Mores CN, Dohm DJ, Lee WJ, Kim HC, Klein TA. Laboratory transmission of Japanese encephalitis, West Nile, and Getah viruses by mosquitoes (Diptera: Culicidae) collected near Camp Greaves, Gyeonggi Province, Republic of Korea 2003. J Med Entomol. 2006;43:1076–81. 10.1603/0022-2585(2006)43[1076:LTOJEW]2.0.CO;2 17017248

