# Supplementary material for: Surveillance for West Nile Virus in Dead Wild Birds, South Korea, 2005–2008
Source: Emerg Infect Dis. 2011 Feb;17(2):299–301. doi: 10.3201/eid1702.100551 (PMC3204754; doi:10.3201/eid1702.100551)
Supplement: Technical Appendix — Reverse Transcription PCR Methods. [file 10-0551-Techapp.pdf]

Article DOI: 10.3201/eid1702.100551

Surveillance for West Nile Virus in Dead Wild Birds, South Korea, 2005–2008

Technical Appendix

Reverse Transcription–PCR Methods

Total RNA was extracted from 50 mg–100 mg of tissue by using the BioRobot M48 (QIAGEN, Valencia, CA, USA) and the MagAttract RNA Cell Mini M48 Kit (QIAGEN). Extracts were eluted into 100 µL nuclease-free water. All samples were extracted and tested in duplicate. A West Nile virus (WNV) control was prepared by extracting RNA from a 100-µL volume containing ten 50% tissue culture infective doses of WNV stock virus. Extracted RNA samples were denatured at 70°C for 10 min.

As reported by Johnson et al. (*1*), reverse transcription–PCR (RT-PCR) was performed by using a 1-step RT-PCR kit (QIAGEN) with 37.5 pmol/L of each of the 2 first-stage primers. Similarly, 2.0 µL of RNase-free water was added to no-template controls that were incubated with diagnostic samples. Reaction tubes were incubated at 45°C for 45 min and 95°C for 11 min, followed by 35 cycles of denaturation at 95°C for 30 s, primer annealing at 55°C for 45 s, and primer extension at 72°C for 60 s.

The nested RT-PCR cycle used similar conditions except for a 5-min primer extension period. For the nested reaction, 1.5 µL of the first-stage amplification product was added to 48.5 µL of a PCR mixture that contained 10 mmol/L Tris-HCl, pH 8.3, 50 mmol/L KCl, 2.0 mmol/L $MgCl_2$, 0.8 mmol/L dNTP pool, 1.0 unit Taq DNA polymerase (QIAGEN), and 37.5 pmol each of the nested primers. Reaction tubes were incubated for 11 min at 95°C, followed by 35 cycles of the cycling conditions described for the first stage. All incubation and amplification

procedures were performed by using a Mastercycler Gradient Thermal Cycler (Eppendorf, Hamburg, Germany).

After RT-PCR, amplification products (5 µL) were analyzed by electrophoresis on a 3% agarose gel containing 0.5 µg/mL ethidium bromide. A 248-bp product indicated that WNV RNA was in the original sample. Following the method of Lanciotti et al. (*2*), we used a 1-step RT-PCR kit with 5 µL of RNA and 50 pmol/L of each primer in a 50-µL reaction volume and the following cycling times and temperatures: 1 cycle at 45°C for 1 h and at 94°C for 3 min and 40 cycles at 94°C for 30 s, 55°C for 1 min, and 68°C for 3 min. After RT-PCR, amplification products (5 µL) were analyzed by gel electrophoresis on a 3% agarose gel containing 0.5 µg/mL ethidium bromide. A 408-bp product indicated that WNV RNA was in the original sample. RNA integrity was confirmed by RT amplification of β-actin mRNA in all samples.

References

1. Johnson DJ, Ostlund EN, Pedersen DD, Schmitt BJ. Detection of North American West Nile virus in animal tissue by a reverse transcription-nested polymerase chain reaction assay. Emerg Infect Dis. 2001;7:739–41. PubMed DOI:10.3201/eid0704.010425

2. Lanciotti RS, Kerst AJ, Nasci RS, Godsey MS, Mitchell CJ, Savage HM, et al. Rapid detection of West Nile virus from human clinical specimens, field-collected mosquitoes, and avian samples by a TaqMan reverse transcriptase-PCR assay. J Clin Microbiol. 2000;38:4066–71. PubMed
